# Effect of different bone cement distributions in percutaneous kyphoplasty on clinical outcomes for osteoporotic vertebral compression fractures: A retrospective study

**DOI:** 10.1097/MD.0000000000033309

**Published:** 2023-03-24

**Authors:** Qichun Song, Yan Zhao, Dong Li, Zhaoying Liu, Yuankai Zhang, Donglong Shang, Zilong Geng, Zhibin Shi, Li-Hong Fan

**Affiliations:** a The Second Affiliated Hospital of Xi’an Jiaotong University, Xi’an, Shaanxi Province, P.R. China; b School of Mechanical Engineering, Xi’an Jiaotong University, Xi’an, Shaanxi, P.R. China.

**Keywords:** bone cement, lumbar vertebra, osteoporotic vertebral compression fractures, percutaneous kyphoplasty

## Abstract

Osteoporotic fractures and their complications are becoming increasingly harmful to the elderly. This study aimed to evaluate the clinical results of connected or unconnected bilateral cement after bilateral percutaneous kyphoplasty (PKP) in patients with osteoporotic vertebral compression fractures (OVCF). The clinical data of 217 patients with single-segment OVCF were retrospectively collected. Patients were allocated into 2 groups according to the bilateral bone cement in the vertebrae was connected or unconnected after surgery. The surgery-related indexes of the 2 groups were compared, including operation time; bone cement injection volume; contact situation between bone cement and the upper and lower endplates of the vertebral body; visual analogue scale (VAS) scores before surgery, 1 week and 1 year after surgery; Oswestry disability index (ODI) before surgery, 1 week and 1 year after surgery; local kyphosis angle (LKA) before surgery, 1 week and 1 year after surgery; postoperative vertebral body height at 1 week and 1 year after surgery; vertebral body height restoration rate (HRR) at 1 week and 1 year after surgery. The follow-up results of all patients were recorded. The postoperative VAS, ODI, vertebral body height, LKA and other indexes of the 2 groups were significantly improved compared with those before the operation (*P* < .05), and there was no significant difference between the 2 groups (*P* > .05). At the same time, there were no significant difference in vertebral body HRR and bone cement leakage rate between the 2 groups (*P* > .05). X-ray examination showed that 21 of 217 patients (21/217, 9.8%) had a refracture of the injured vertebral body, including 16 cases (16/121, 13.2%) in the unconnected group and 5 cases (5/96, 5.2%) in the connected group (*P* < .05). Adjacent vertebrae fractures occurred in 25 cases (25/217, 11.5%), while 19 cases (19/121, 15.7%) were in the unconnected group and 6 cases (6/96, 6.3%) were in the connected group (*P* < .05). PKP has a good therapeutic effect on OVCF no matter whether the bilateral bone cement is connected or not. However, if the bilateral cement inside the vertebra was connected, the risk of recollapse of the injured vertebrae and the new fracture of adjacent vertebrae could be reduced.

## 1. Introduction

Vertebral compression fracture is one of the main health problems in the elderly. The annual incidence of vertebral compression fractures is 10/1000 for women, and 5/1000 for men, which has been one of the main causes of poor quality of life and a heavy burden on the national health care budget.^[1,2]^ The elderly, especially those with osteoporosis, are more likely to suffer from osteoporotic vertebral compression fractures (OVCF). Particularly among women, there are approximately 1.5 million OVCF patients each year.^[3,4]^ At present, there are surgical and nonsurgical methods for the treatment of OVCF. nonoperative methods include taking painkillers, wearing back braces, and bed rest to improve functional status and preventing future fractures of other vertebral bodies. However, these methods have limited efficacy and severe side effects, such as thrombosis and lung infection.^[5,6]^ At the same time, elderly patients with osteoporosis have a high risk of surgical accidents, so the traditional open surgery is generally not recommended.^[7]^ Therefore, minimally invasive spinal surgery has been widely used for vertebral body expansion in OVCF patients, including percutaneous kyphoplasty (PKP) and percutaneous vertebroplasty.^[8]^ Previous studies showed that both PKP and percutaneous vertebroplasty could achieve satisfactory clinical effects in OVCF treatment, while PKP has a lower cement leakage rate, better kyphotic angle and better vertebral height recovery.^[9–11]^ Therefore, more and more surgeons put PKP to the first position in treating OVCF patients.

The bone cement used in PKP is made of viscous polymethyl methacrylate (PMMA). The cytotoxic and febrile effects of PMMA can damage the bone peripheral nerves and stabilize micro-movements by strengthening the vertebral body.^[12]^ However, PMMA has nondegradability and high biomechanical stress, which might cause recollapse.^[13]^ In addition, the actual position of cement in the vertebral body may be affected by the differences of surgical techniques, different choices of dilators, and changes in the anatomical structure of the puncture vertebral body. For example, when performing PKP surgery on the lateral pedicle, the bone cement on both sides may or may not be connected. At the same time, excessive injection of cement may cause some biomechanical changes. This indicates that the most appropriate intravertebral cement volume should be used to obtain the best bone cement distribution in the vertebral body to achieve the best postoperative results. Some studies have confirmed that proper cement distribution and a small amount of cement can achieve good surgical results.^[14,15]^ What more, several recent studies have shown that the reduction of PMMA contact with the upper and lower endplates is a risk factor for the recollapse of the fractured vertebral body. Therefore, better controlling the distribution of PMMA during surgery could reduce the risk of recollapse after kyphoplasty. If the bone cement fully contacts the upper and lower endplates, it would better restore the strength of the vertebral body, maintain the height of the vertebral body, reduce the risk of vertebral body recollapse and long-term pain.^[16–18]^ But so far, few people have studied the effect of whether the 2 sides of bone cement are connected during bilateral PKP on the postoperative efficacy of patients. Therefore, this study analyzed the influence of whether the 2 sides of bone cement are connected on the postoperative efficacy of patients through retrospective research so as to provide surgeons with more effective surgical methods and minimize complications.

## 2. Methods

### 2.1. Patients and grouping

This retrospective investigation collected the data of the OVCF patients who received bilateral PKP treatment in The Second Affiliated Hospital of Xi’an Jiao tong University from May 2017 to November 2020, which included 217 patients who met the inclusion criteria. The inclusion criteria were as follows: A single-level OVCF; 15% < collapse < 60%; (3) 1 day < Symptom duration < 6 weeks; Visual Analogue Scale (VAS) > 5; The score of bone mineral density (BMD) < −2.5; age > 55years old; with a complete follow-up of > 1 years. The exclusion criteria were as follows: Inability to give informed consent; Poor general physical state; Caused by malignant disease; The pedicles or the back wall of the vertebra was broken; Associated with spinal stenosis or disc herniation; Fractures with lower limb symptoms. This study was approved by the Ethics Committee of Xi’an Jiaotong University, and all participants signed the informed consent to the clinical study.

**Figure 1.**
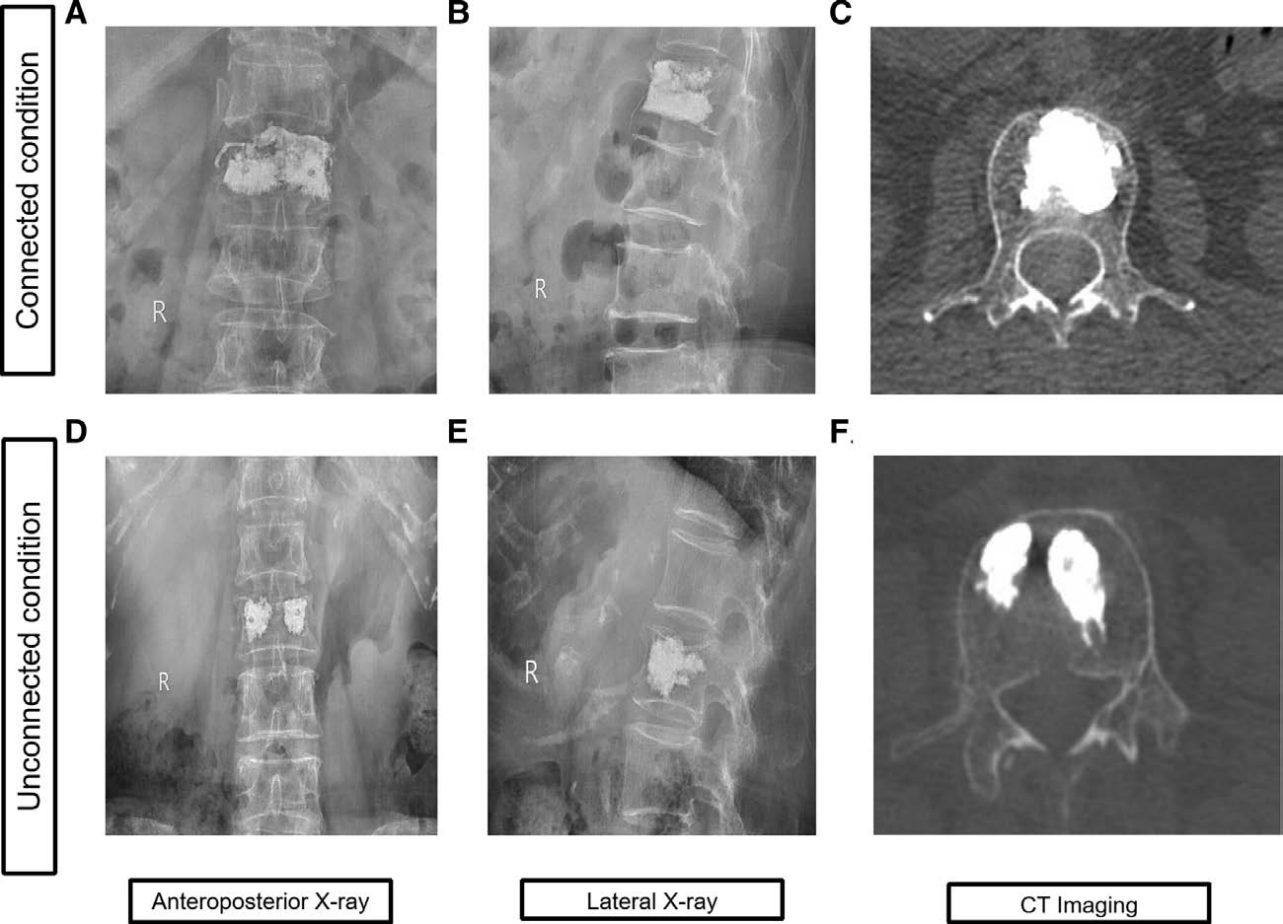
Postoperative X-ray and CT imaging. A and B, the bilateral cement-connected group; D and E, the bilateral cement-unconnected group; C, the CT imaging of the bilateral cement connected group, bone cement was injected from both sides and connected in the center of the vertebra; F, the CT imaging of the bilateral cement unconnected group, bone cement was injected from both sides and did not connect in the center of the vertebra. CT = computer tomography.

Moreover, the patients were divided into the bone-cement connected group and unconnected group according to the X-rays taken 1 week after the operation. Connected group: X-ray front and lateral radiographs show that the bone cement on both sides are connected. Unconnected group: X-ray front and lateral radiographs show that the bone cements on both sides are not connected. Thin-slice CT scan (Fig. 1) was used when it is difficult to determine the classification of the patients.

### 2.2. Basic data

Age, gender ratio, BMD, location of fractured vertebra, the interval between symptom onset and PKP, preoperative vertebral body height, Oswestry disability index (ODI), local kyphosis angle (LKA) and preoperative VAS of the 2 groups were recorded.

Unconnected group comprised 48 (48/121, 39.7%) males and 73 (73/121, 60.3%) females aged from 56 to 81 (average,70.4 ± 9.2) years, with an average BMD of −3.0 ± 0.7. Thoracic spine fractures were found in 49 (49/121, 40.5%) patients and lumbar spine fractures were identified in 72 (72/121, 59.5%) patients. The average follow-up time was 13.6 ± 1.3 months. The preoperative VAS score was 8.1 ± 1.1, preoperative ODI was 55.1 ± 5.0, preoperative vertebral body height was 2.0 ± 0.5 cm and the LKA was 14.5 ± 6.2°. The interval between symptom onset and PKP was 18.9 ± 6.0 (1–31) days (Table 1).

**Table 1:** Patient characteristics and general information.

Items	Unconnected group (n = 121)	Connected group (n = 96)	*χ*^2^ or *T* value	*P* value
Sex			0.0204	.8866
Female	73 (60.3%)	57 (59.4%)		
Male	48 (39.7%)	39 (40.6%)		
Age (yr)			0.0932	.9545
<60	37 (30.6%)	28 (29.1%)		
60–79	53 (43.8%)	44 (45.8%)		
≥80	31 (25.6%)	24 (25.0%)		
Average age (yr)	70.4 ± 9.2	69.9 ± 7.8	0.4249	.6713
BMD (*T* score)			0.4387	.8031
<−4.5	24 (19.8%)	20 (20.8%)		
−4.5 to −3.5	43 (35.5%)	31 (32.3%)		
−3.4 to −2.5	54 (44.7%)	45 (46.9%)		
Average BMD	−3.0 ± 0.7	−3.1 ± 0.6	1.1124	.2672
Fractured vertebra			0.0854	.7700
Thoracic vertebra	49 (40.5%)	37 (38.5%)		
Lumber vertebra	72 (59.5%)	59 (61.5%)		
Preoperative VAS, mean ± SD	8.1 ± 1.1	7.9 ± 1.0	1.3844	.1677
Preoperative ODI, mean ± SD	55.1 ± 5.0	53.9 ± 5.2	1.7251	.0859
The interval between symptom onset and PKP, days, mean ± SD	18.9 ± 6.0	20.1 ± 5.8	1.4849	.1390
Preoperative vertebral body height, cm, mean ± SD	2.0 ± 0.5	2.1 ± 0.4	1.5957	.1120
Preoperative local kyphosis angle (LKA), °, mean ± SD	14.5 ± 6.2	15.2 ± 6.6	0.8028	.4230

BMD = bone mineral density, LKA = local kyphosis angle, ODI = oswestry disability index, PKP = percutaneous kyphoplasty, SD = standard deviation, VAS = visual analogue scale.

Connected group comprised 39 (39/96, 40.6%) males and 57 (57/96, 59.4%) females aged from 57 to 83 (average,69.9 ± 7.8) years, with an average BMD of −3.1 ± 0.6. Thoracic spine fractures were found in 37 (37/96, 38.5%) patients and lumbar spine fractures were recognized in 59 (59/96, 61.5%) patients. The average follow-up time was 13.1 ± 1.4 months. The preoperative VAS score was 7.9 ± 1.0, preoperative ODI was 53.9 ± 5.2, preoperative vertebral body height was 2.1 ± 0.4 cm and the LKA was 15.2 ± 6.6°. The interval between symptom onset and PKP was 20.1 ± 5.8 (1–37) days (Table 1).

### 2.3. Surgical procedures

The PKP procedure was performed by the same senior physician. Place the patient in the prone position, perform local anesthesia, and install a C-arm for guidance. Using a small incision, the working cannula is inserted into the vertebral body through the bilateral pedicle approach. Through the working casing, the drill bit is advanced, creating a channel for the balloon. Depending on the size of the vertebral body, a balloon with a diameter of 15 or 20 mm is used. The balloon is inserted into the cancellous bone of the vertebral body, and the contrast agent iohexol is injected through a high-pressure pump to slowly expand the balloon. Once a satisfactory Cobb angle and vertebral height relative to the preoperative level have been determined by C-arm radiography, the contrast agent is extracted, and the balloon is deflated and removed. The same operation was performed on the other side of the vertebral body. Subsequently, PMMA was injected into the vertebral body under low pressure to fill the gap, and as far as possible to make the PMMA and the endplate fully contact. The procedure should be performed carefully to prevent the bone cement leakage. Finally, the working sleeve is removed and the skin entrance closed with a single suture. The operation time and bone cement injection volume were recorded and compared.

### 2.4. Postoperative treatment and follow-up

The patient needs to rest in bed for 24 hours. Orthosis brace was applied for 3 months after the surgery and antiosteoporosis therapy lasted for a minimum of 1 year. The patients were followed up to evaluate effects and complications by senior surgeons (LHF and ZBS) in clinics or via telephone or email every month at least for 1 year.

### 2.5. Postoperative outcome measurement

The surgery-related indexes of the 2 groups were compared, including operation time; bone cement injection volume; contact situation between bone cement and the upper and lower endplates of the vertebral body; VAS scores before surgery, 1 week and 1 year after surgery; ODI before surgery, 1 week and 1 year after surgery; LKA before surgery, 1 week and 1 year after surgery; postoperative vertebral body height at 1 week and 1 year after surgery; vertebral body height restoration rate (HRR) at 1 week and 1 year after surgery; The follow-up results of all patients were recorded.

The degree of focal back pain was assessed by the VAS (0 = no pain, 10 = most severe pain). ODI was applied to assess the improvement in the ability to daily function. It assessed the aspects including pain intensity, lifting, walking, sleeping social life, etc.^[19]^ 0% stands for minimal disability while 100% stands for extreme disability and the patients may be in bed bound or with exaggerating symptoms.

**Figure 2.**
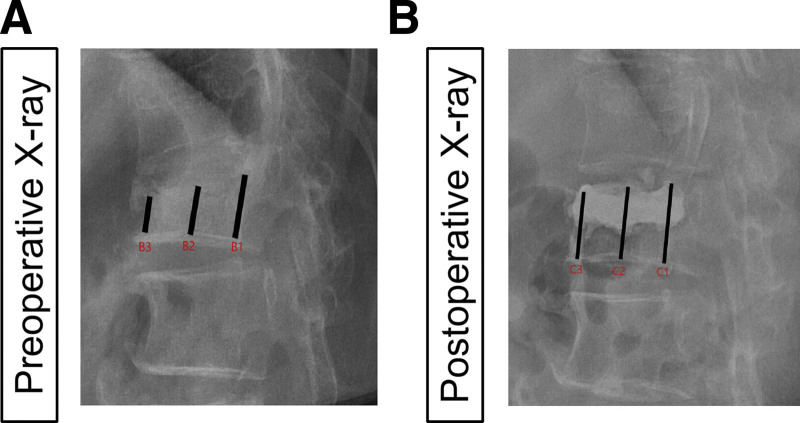
Measurement of vertebral height. PFH(B2) and PRH(C2) are equal to (B1 + B3)/2 and (C1 + C3)/2, respectively; EOH was accepted as the average of the height of the normal vertebral bodies above and below the fractured vertebra; HRR can be calculated as: HRR = (PRH-PFH)/EOH X 100%. EOH = estimated original vertebral body height, HRR = height restoration rate, PFH = preoperative fractured vertebral body height, PRH = postoperative restored vertebral body height.

**Figure 3.**
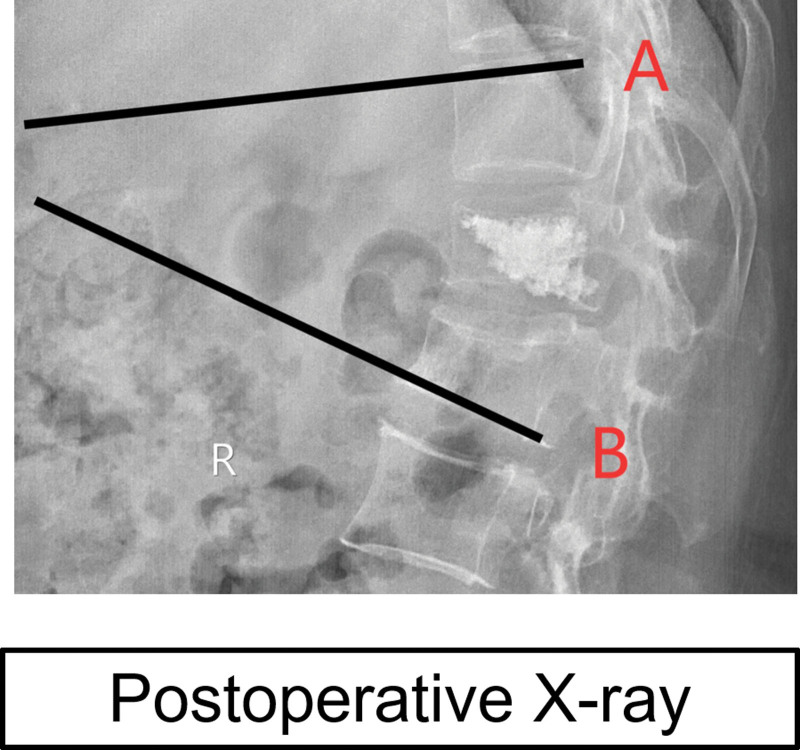
Measure the Local kyphosis angle. Line A is the parallel line of the upper end plate of the first vertebra above the fracture vertebra and Line B is the parallel line of the lower end plate of the first vertebra below the fracture vertebra. The LKA is the angle between A and B. LKA = local kyphosis angle.

Radiological parameters included: estimated original vertebral body height (EOH), preoperative fractured vertebral body height (PFH), postoperative restored vertebral body height (PRH), HRR and LKA, which were assessed by plain X-ray. EOH was accepted as the average of the height of the normal vertebral bodies above and below the fractured vertebra. PFH (B2) and PRH (C2) are the mean values of the vertebral posterior edge (B1/C1) and the leading edge (B3/C3) (Fig. 2). HRR represented the percentage restored from the preoperative height, it can be calculated as: HRR = (PRH-PFH)/EOH. The LKA was defined as the angle formed by lines drawn parallel to the upper end plate of the first vertebra above the fractured vertebra and the lower end plate of the first vertebra below the fractured vertebra (Fig. 3).

Cement leakage is defined as the presence of any extra vertebral high cement signal observed by X-rays. New fractures of 2 adjacent vertebral bodies of the injured vertebral body are defined as adjacent vertebral body fractures.

### 2.6. Statistical analysis

Statistical analysis was conducted using IBM SPSS 24.0 (IBM SPSS Inc., Chicago). The normally distributed measurement data were expressed as mean ± standard deviation ($\bar{x}\;\;\pm \;S$) and percentage and compared using the *t* test. The enumeration data were expressed as percentages (%) and compared using the chi-square test. The statistical significance was set as *P* < .05.

## 3. Results

### 3.1. Basic information of patients

The basic data, including mean age, gender ratio, BMD, location of fractured vertebra, the interval between symptom onset and PKP, preoperative vertebral body height, preoperative ODI, preoperative LKA and preoperative VAS of the 2 groups, were not significantly different between the 2 groups (*P* > .05) (Table 1).

### 3.2. Comparison of surgery-related indexes

All patients successfully received PKP. The postoperative imaging showed that the injection of bone cement could effectively restore the height of the vertebral body and the local kyphotic angle.

The operation time was 36.2 ± 5.8 minutes in the unconnected group and 37.1 ± 6.3 minutes in connected group. The cement injection volume was 3.8 ± 1.2 mL in the unconnected group and 4.0 ± 1.1 mL in the connected group. There was no significant difference in operation time and bone cement injection volume between the 2 groups (*P* > .05) (Table 2).

**Table 2:** The comparison of surgery-related indexes between the 2 groups.

Items	Unconnected group (n = 121)	Connected group (n = 96)	*χ*^2^ or *T* value	*P* value
Operative time (min), mean ± SD	36.2 ± 5.8	37.1 ± 6.3	1.9027	.2757
Bone cement injection volume (ML), mean ± SD	3.8 ± 1.2	4.0 ± 1.1	1.2649	.2073
Position of bone cement and end plate			0.8036	.6691
Contact the upper and lower end plate	79 (65.3%)	57 (59.4%)		
Contact the upper or lower end plate	31 (25.6%)	29 (30.1%)		
The central vertebral bodies	11 (9.1%)	10 (10.4%)		

SD = standard deviation.

In the unconnected group, there were 79 (79/121, 65.3%) cases were in contact with the upper and lower endplates, 31 (25.6%) cases were only in contact with the upper or lower endplates, and 11 (11/121, 9.1%) cases were not in contact with the upper or lower endplates and located in the center of the vertebral body. For the connected group, there were 57 (57/96, 59.4%) cases were in contact with the upper and lower end plates, 29 (30.1%) cases were only in contact with the upper or lower endplate, and 10 (10/96, 10.4%) cases were not in contact with the upper or lower endplates and located in the center of the vertebral body. No significant differences were found in contact situation among bone cement and the upper and lower endplates of the vertebral body between the 2 groups (*P* > .05) (Table 2).

### 3.3. Comparison of the clinical outcomes between the 2 groups

No significant differences were found in preoperative VAS or ODI between the 2 groups (*P* > .05). In the unconnected group, the average preoperative VAS score was 8.1 ± 1.1, which significantly decreased to 2.0 ± 0.7 at 1 week after operation and 1.9 ± 0.5 at the 1 year after operation (*P* < .05). In the connected group, the average preoperative VAS score was 7.9 ± 1.0, which significantly decreased to 2.1 ± 0.9 at 1 week after operation and 1.8 ± 0.6 at the 1 year after operation (*P* < .05). In the unconnected group, the average preoperative ODI was 55.1 ± 5.0, which significantly decreased to 21.5 ± 2.3 at 1 week after operation and 20.1 ± 1.9 at the 1 year after operation (*P* < .05). In the connected group, the average preoperative ODI was 53.9 ± 5.2, which significantly decreased to 20.9 ± 2.7 at 1 week after operation and 19.6 ± 2.1 at the 1 year after operation (*P* < .05). There was no significant difference between the 2 groups at different time points, as shown in Table 3.

**Table 3:** Comparison of the clinical outcomes between 2 groups.

Items	Unconnected group (n = 121)	Connected group (n = 96)	*T* value	*P* value
VAS, mean ± SD				
Preoperative	8.1 ± 1.1	7.9 ± 1.0	1.3844	.1677
1 wk	2.0 ± 0.7*	2.1 ± 0.9*	0.9208	.3582
1 yr	1.9 ± 0.5*	1.8 ± 0.6*	1.3389	.1820
ODI, mean ± SD				
Preoperative	55.1 ± 5.0	53.9 ± 5.2	1.7251	.0859
1 wk	21.5 ± 2.3*	20.9 ± 2.7*	1.7668	.0787
1 yr	20.1 ± 1.9*	19.6 ± 2.1*	1.4.700	.1430

ODI = oswestry disability index, SD = standard deviation, VAS = visual analogue scale.

* Compared with preoperation, *P* < .05.

### 3.4. Comparison of the radiological results between the 2 groups

The patients in the 2 groups showed significant restoration of vertebral body height at 1 week and 1 year after operation (*P* < .05). Although at the 1-year follow-up, height of fracture vertebra loss occurred in both groups, no significant differences were found between the 2 groups (*P* > .05). The HRR (after operation 1 week/1 year follow-up) was 20.5 ± 7.3%, 17.3 ± 6.7% in the unconnected group and 21.2 ± 8.2%, 18.6 ± 6.1% in the connected group, and there was no significant difference in HRR between the 2 groups (*P* > .05) (Table 4).

**Table 4:** Comparison of the radiological results between 2 groups.

Items	Unconnected group (n = 121)	Connected group (n = 96)	T value	*P* value
PFH, mean ± SD, cm	2.0 ± 0.5	2.1 ± 0.4	1.5957	.1120
PRH, mean ± SD, cm				
1 wk	2.6 ± 0.7*	2.5 ± 0.8*	0.9810	.3277
1 yr	2.3 ± 0.6*	2.4 ± 0.7*	1.1324	.2587
HRR, mean ± SD, %				
1 wk	20.5 ± 7.3	21.2 ± 8.2	0.2587	.5073
1 yr	17.3 ± 6.7	18.6 ± 6.1	1.4765	.1413
LKA, mean ± SD, °				
Preoperative	14.5 ± 6.2	15.2 ± 6.6	0.8028	.4230
1 wk	7.3 ± 2.9*	6.8 ± 3.1*	1.2235	.2225
1 yr	10.1 ± 4.0*	11.0 ± 4.3*	1.5924	.1128

HRR = height restoration rate, LKA = local kyphosis angle, PFH = preoperative fractured vertebral body height, PRH = postoperative restored vertebral body height, SD = standard deviation.

* Compared with preoperation, *P* < .05.

The LKA (preoperatively/after operation 1 week/1 year follow-up) was 14.5 ± 6.2°/7.3 ± 2.9°/10.1 ± 4.0° in the unconnected group and 15.2 ± 6.6°/6.8 ± 3.1°/11.0 ± 4.3°in the connected group (Table 4). The mean LKA improved significantly in these 2 groups at 1 week and 1 year after operation (*P* < .05). There was no significant difference between the 2 groups at all the time points in terms of LKA (*P* > .05).

### 3.5. Surgical results and complications

According to the last X-ray radiographs of the spine, there were 20 cases (20/217, 9.2%) of bone cement leakage in all the patients, 9 cases (9/121, 7.4%) in the unconnected group and 11 cases (11/96, 11.5%) in the connected group (*P* > .05). All the cases of cement leakage into the disc space or anterior vertebra showed no clinical symptom. The number for refracture of the injured vertebrae were 21 cases (21/217, 9.8%) in all the patients, 16 cases (16/121, 13.2%) in the unconnected group and 5 cases (5/96, 5.2%) in the connected group. Among all patients, 25 cases (25/217, 11.5%) had adjacent vertebral fractures, 19 cases (19/121, 15.7%) in the unconnected group and 6 cases (6/96, 6.3%) in the connected group. There were significant differences in the incidence of refracture of the injured vertebrae and new fracture of adjacent vertebral body between the 2 groups (*P* < .05) (Table 5).

**Table 5:** The comparison of complications between the 2 groups, n (%).

Items	Unconnected group (n = 121)	Connected group (n = 96)	*χ*^2^ value	*P* value
Cement leakage	9 (7.4%)	11 (11.5%)	1.0340	.3092
Refracture of injured vertebrae	16 (13.2%)	5 (5.2%)	3.9339	.0473*
Adjacent vertebral fracture	19 (15.7%)	6 (6.3%)	4.6921	.0303*

* There was statistical difference when *P* < .05.

## 4. Discussion

Vertebral compression fractures often occur in the thoracolumbar vertebrae of the spine. It is one of the common complications of osteoporosis, especially in the elderly and postmenopausal women.^[1,20]^ The patient not only has persistent pain at the fracture site, but also it is accompanied by loss of vertebral body height, spinal instability and kyphosis, which seriously affect the quality of life.^[21]^ However, with the widespread application of PKP technology, the increased risk of adjacent vertebral fractures, recollapse of the strengthened vertebral body, and high economic costs have gradually attracted people attention.^[22–24]^ Therefore, how to use PKP more efficiently to treat OVCF, reduce the suffering of patients, obtain a better quality of life and reduce the economic burden of national medical insurance is the focus of our research.

Chen et al showed that unilateral and bilateral PKP can obtain similar good clinical and radiological results through 8 eligible meta-analyses.^[25]^ Zhang et al conducted a retrospective study and found that unilateral and bilateral PKP could improve the clinical symptoms of OVCF, and the vertebral body height could be effectively restored within at least 18 months after surgery.^[26]^ Michael et al revealed that a large filling volume may not be the best biomechanical configuration, overfilling might cause the vertebral body to be more sensitive to bone cement.^[27]^ That is to say, the increase of bone cement volume may not only increase the risk of bone cement leakage, but also increase the risk of fracture of adjacent vertebral bodies. It is not an appropriate method to obtain wide distribution of bone cement by increasing excessive bone cement volume.^[28]^

Many previous literatures also have confirmed that bilateral puncture did not significantly increase the risk of complications such as bone cement leakage and nerve injury as long as the puncture route was strictly followed.^[29,30]^ Several studies have investigated the relationship between the distribution of bone cement in the sagittal position and the surgical outcome.^[15,18]^ However, we found that few studies have documented the effect of bilateral cement connected on long-term outcomes. Therefore, it may be more meaningful to study the distribution of bone cement on the coronal plane on the anteroposterior radiographs for the analysis of surgical efficacy.

In this study, there were no significant differences in basic data and surgery-related indexes between the 2 groups. However, the significant difference in the risk of recollapse of the injured vertebra and adjacent vertebral fractures between the 2 groups at 1 year after surgery attracted our attention. In this study, the mean follow-up time of patients was 17.4 ± 4.6 (6–24) months. Our study found that if the bilateral cement inside the vertebra was connected, the risk of refracture of the vertebra will be reduced, there were significant differences in the incidence of refracture of the injured vertebrae and new fracture of adjacent vertebral body between the 2 groups (*P* < .05). The number of refractures of the injured vertebrae is 21 cases (21/217, 9.8%) in all the patients, 16 cases (16/121, 13.2%) in the unconnected group and 5 cases (5/96, 5.2%) in the connected group (*P* < .05). Among all patients, 25 cases (25/217, 11.5%) had adjacent vertebral fractures, including 19 cases (19/121, 15.7%) in the unconnected group and 6 cases (6/96, 6.3%) in the connected group (*P* < .05). The refracture of the injured vertebra and new fracture of the adjacent vertebra are multifactorial, and may be related to the degree of osteoporosis, daily activities, and distribution of bone cement. The distribution of bone cement is an important cause of recurrent vertebral fractures, especially the uneven stress caused by the asymmetric distribution in the coronal plane.^[31,32]^ We consider that when the bone cement on both sides are connected, the intravertebral bone cement as a whole can not only increase the strength of the injured vertebral body, but also better resist the stress caused by the body rotation, thus reducing the risk of the injured vertebral body refracture and new fractures of the adjacent vertebral bodies. At the same time, some literatures pointed out that the noncontact of cement and end plate was a risk factor for collapse again.^[16,18,33]^ In our study, without causing bone cement leakage, the bone cement should be distributed evenly and symmetrically as far as possible, and in contact with the upper and lower endplates. Fortunately, the distribution of bone cement was similar between the 2 groups.

At the same time, bone cement leakage of the intervertebral disc is an independent risk factor for adjacent vertebral fracture, the exuded bone cement can cause persistent high stress on the intervertebral disc.^[34,35]^ In our study, a total of 20 (20/217, 9.2%) patients had bone cement leakage, including 9 cases (9/121, 7.4%) of unconnected group and 11 cases (11/96, 11.5%) of connected group. Although the rate of bone cement leakage was higher (11/96, 11.5%) in the connected group than in the unconnected group, there was no statistical difference between the 2 groups (*P* > .05). The bilateral cement connection has high rate of cement leakage, which could be explained by that when the bilateral cement connection is pursued during the surgery, the cement may leak into the disc due to the end plate fracture or the end plate crack, increasing the risk of cement leakage. Therefore, we recommend that the operation of bilateral bone cement connection should be completed under the continuous radiography by C-arm to avoid bone cement leakage as much as possible.

There are some limitations in this current study. First, this is a retrospective study with a small sample. The clinical results in this study did not cover a wide range of terminal results. Therefore, single-center or multi-center prospective studies are needed to provide stronger evidence for clinical practice. In addition, our study only analyzed the postoperative clinical results of different bone cement distribution modes, but did not detect the changes in the vertebral body biological stress under different bone cement distribution modes, which should be supplemented by finite element analysis or biomechanical analysis in subsequent studies to draw more accurate conclusion. Finally, our study only focused on the fractures of the injured vertebrae and adjacent vertebrae, other vertebral fractures were not included, which also need to be improved in subsequent studies.

## 5. Conclusion

Whether the bilateral bone cement is connected or not, bilateral PKP treatment of OVCF could provide better vertebral strength, vertebral HRR and LKA, which could reduce patient suffering and improve patient survival quality. However, in bilateral PKP surgery, if the bilateral cement inside the vertebra was connected, the risk of refracture of the injured vertebrae and the new fracture of the adjacent vertebral body might be reduced to achieve better operation results.

## Author contributions

**Conceptualization:** Qichun Song, Li-Hong Fan.

**Data curation:** Qichun Song, Yan Zhao, Dong Li, Zhaoying Liu, Donglong Shang, Zilong Geng.

**Formal analysis:** Dong Li, Zhaoying Liu, Zilong Geng.

**Funding acquisition:** Qichun Song, Li-Hong Fan.

**Investigation:** Yan Zhao, Dong Li, Donglong Shang.

**Methodology:** Yan Zhao, Yuankai Zhang.

**Project administration:** Li-Hong Fan.

**Software:** Zhaoying Liu, Donglong Shang, Zilong Geng.

**Supervision:** Zhibin Shi, Li-Hong Fan.

**Writing – original draft:** Qichun Song, Yan Zhao.

**Writing – review & editing:** Qichun Song, Yan Zhao, Yuankai Zhang, Zhibin Shi, Li-Hong Fan.
